# Supplementary material for: Evaluating multi-locus phylogenies for species boundaries determination in the genus Diaporthe
Source: PeerJ. 2017 Mar 28;5:e3120. doi: 10.7717/peerj.3120 (PMC5372842; doi:10.7717/peerj.3120)
Supplement: Table S1 [file peerj-05-3120-s001.docx]

| **Species** | **Strain** | **Gen Bank Accession Number** | | | | |
| --- | --- | --- | --- | --- | --- | --- |
|  |  | **ITS** | **CAL** | **HIS** | **TEF1** | **TUB** |
| *Diaporthe acaciigena* | CBS 129521 = CPC 17622 (ex-type) | KC343005 | KC343247 | KC343489 | KC343731 | KC343973 |
| *Diaporthe acerina* | CBS 137.27 | KC343006 | KC343248 | KC343490 | KC343732 | KC343974 |
| *Diaporthe alleghaniensis* | CBS 495.72 (isotype) = ATCC 24097 (ex-type) | KC343007 | KC343249 | KC343491 | KC343733 | KC343975 |
| *Diaporthe alnea* | CBS 146.46 | KC343008 | KC343250 | KC343492 | KC343734 | KC343976 |
|  | CBS 159.47 | KC343009 | KC343251 | KC343493 | KC343735 | KC343977 |
| *Diaporthe ambigua* | CBS 114015 (epitype ) = STE-U 2657 = CPC 2657 (ex-epitype) | KC343010 | KC343252 | KC343494 | KC343736 | KC343978 |
|  | CBS 117167 = STE-U 5414 = CPC 5414 | KC343011 | KC343253 | KC343495 | KC343737 | KC343979 |
| *Diaporthe ampelina* | CBS 111888; ATCC 48153; STE-U 2673 = CPC 2673 | KC343016 | KC343258 | KC343500 | KC343742 | KC343984 |
| *Diaporthe amygdali* | CBS 126679 (epitype ) | KC343022 | KC343264 | KC343506 | KC343748 | KC343990 |
|  | CBS 111811 = STE-U 2632 = CPC 2632 | KC343019 | KC343261 | KC343503 | KC343745 | KC343987 |
| *Diaporthe anacardii* | CBS 720.97 (epitype) | KC343024 | KC343266 | KC343508 | KC343750 | KC343992 |
| *Diaporthe angelicae* | CBS 111592 = AR3776 (ex-epitype) | KC343027 | KC343269 | KC343511 | KC343753 | KC343995 |
|  | CBS 123215; Ph-C133/1 | KC343028 | KC343270 | KC343512 | KC343754 | KC343996 |
| *Diaporthe arctii* | CBS 136.25 | KC343031 | KC343273 | KC343515 | KC343757 | KC343999 |
| *Diaporthe arecae* | CBS 161.64 (isotype of *Subramanella arecae*) | KC343032 | KC343274 | KC343516 | KC343758 | KC344000 |
|  | CBS 535.75 | KC343033 | KC343275 | KC343517 | KC343759 | KC344001 |
| *Diaporthe arengae* | CBS 114979 (holotype) = HKUCC 5527 (ex-type) | KC343034 | KC343276 | KC343518 | KC343760 | KC344002 |
| *Diaporthe aspalathi* | CBS 117169 = STE-U 5428 = CPC 5428 (ex-epitype) = R412AY | KC343036 | KC343278 | KC343520 | KC343762 | KC344004 |
|  | CBS 117168 = STE-U 5420 = CPC 5420 | KC343035 | KC343277 | KC343519 | KC343761 | KC344003 |
| *Diaporthe australafricana* | CBS 111886 = STE-U 2676 = CPC 2676 (ex-epitype) | KC343038 | KC343280 | KC343522 | KC343764 | KC344006 |
|  | CBS 113487 = STE-U 2655 = CPC 2655 (holotype) | KC343039 | KC343281 | KC343523 | KC343765 | KC344007 |
| *Diaporthe batatas* | CBS 122.21 | KC343040 | KC343282 | KC343524 | KC343766 | KC344008 |
| *Diaporthe beckhausii* | CBS 138.27 | KC343041 | KC343283 | KC343525 | KC343767 | KC344009 |
| *Diaporthe bicincta* | CBS 121004 = DP 0659 | KC343134 | KC343376 | KC343618 | KC343860 | KC344102 |
| *Diaporthe brasiliensis* | CBS 133183 = LGMF924 = CPC 20300 (ex-epitype) | KC343042 | KC343284 | KC343526 | KC343768 | KC344010 |
|  | LGMF926 = CPC 20302 | KC343043 | KC343285 | KC343527 | KC343769 | KC344011 |
| *Diaporthe carpini* | CBS 114437 = UPSC 2980 | KC343044 | KC343286 | KC343528 | KC343770 | KC344012 |
| *Diaporthe caulivora* | CBS 127268 (neotype) = Dpc1 (ex-neotype) | KC343045 | KC343287 | KC343529 | KC343771 | KC344013 |
|  | CBS 178.55 = ATCC 12048 = Alfaro 243 | KC343046 | KC343288 | KC343530 | KC343772 | KC344014 |
| *Diaporthe celastrina* | CBS 139.27 (ex-type) | KC343047 | KC343289 | KC343531 | KC343773 | KC344015 |
| *Diaporthe chamaeropis* | CBS 454.81 | KC343048 | KC343290 | KC343532 | KC343774 | KC344016 |
|  | CBS 753.70 | KC343049 | KC343291 | KC343533 | KC343775 | KC344017 |
| *Diaporthe cinerascens* | CBS 719.96 | KC343050 | KC343292 | KC343534 | KC343776 | KC344018 |
| *Diaporthe citri* | CBS 199.39 | KC343051 | KC343293 | KC343535 | KC343777 | KC344019 |
|  | CBS 230.52 | KC343052 | KC343294 | KC343536 | KC343778 | KC344020 |
| *Diaporthe convolvuli* | CBS 124654 = DP 0727 | KC343054 | KC343296 | KC343538 | KC343780 | KC344022 |
| *Diaporthe crataegi* | CBS 114435 = UPSC 2938 | KC343055 | KC343297 | KC343539 | KC343781 | KC344023 |
| *Diaporthe crotalariae* | CBS 162.33 (type) | KC343056 | KC343298 | KC343540 | KC343782 | KC344024 |
| *Diaporthe cuppatea* | CBS 117499 (holotype) = STE-U 5431 = CPC 5431 (ex-type) | KC343057 | KC343299 | KC343541 | KC343783 | KC344025 |
| *Diaporthe cynaroidis* | CBS 122676 (holotype) = CMW 22190 = CPC 13180 (ex-type) | KC343058 | KC343300 | KC343542 | KC343784 | KC344026 |
| *Diaporthe decedens* | CBS 109772 = AR 3459 | KC343059 | KC343301 | KC343543 | KC343785 | KC344027 |
|  | CBS 114281 = UPSC 2957 | KC343060 | KC343302 | KC343544 | KC343786 | KC344028 |
| *Diaporthe detrusa* | CBS 109770 = AR 3424 | KC343061 | KC343303 | KC343545 | KC343787 | KC344029 |
|  | CBS 114652 = UPSC 3371 | KC343062 | KC343304 | KC343546 | KC343788 | KC344030 |
| *Diaporthe elaeagni* | CBS 504.72 | KC343064 | KC343306 | KC343548 | KC343790 | KC344032 |
| *Diaporthe endophytica* | CBS 133811 (type) = LGMF916 = CPC 20292 (ex-type) | KC343065 | KC343307 | KC343549 | KC343791 | KC344033 |
|  | LGMF928 = CPC 20304 | KC343068 | KC343310 | KC343552 | KC343794 | KC344036 |
| *Diaporthe eres* | CBS 439.82 (isotype of *Phomopsis cotoneastri*) = BBA P-407 = IMI 162181a | KC343090 | KC343332 | KC343574 | KC343816 | KC344058 |
|  | CBS 101742 | KC343073 | KC343315 | KC343557 | KC343799 | KC344041 |
|  | CBS 109767 (type) = AR 3538 ex WJ 1643 | KC343075 | KC343317 | KC343559 | KC343801 | KC344043 |
|  | CBS 113470 = DAOM 226800 | KC343146 | KC343388 | KC343630 | KC343872 | KC344114 |
|  | CBS 116953 = NZ-26 | KC343147 | KC343389 | KC343631 | KC343873 | KC344115 |
|  | CBS 200.39 | KC343151 | KC343393 | KC343635 | KC343877 | KC344119 |
| *Diaporthe eugeniae* | CBS 444.82 | KC343098 | KC343340 | KC343582 | KC343824 | KC344066 |
| *Diaporthe fibrosa* | CBS 109751 = AR 3425 | KC343099 | KC343341 | KC343583 | KC343825 | KC344067 |
|  | CBS 113830 = UPSC 2117 | KC343100 | KC343342 | KC343584 | KC343826 | KC344068 |
| *Diaporthe foeniculacea* | CBS 123208 (holotype of *D. neotheicola*) = Di-C004/5 (ex-type of *D. neotheicola*) | KC343104 | KC343346 | KC343588 | KC343830 | KC344072 |
|  | CBS 111553 | KC343101 | KC343343 | KC343585 | KC343827 | KC344069 |
|  | CBS 187.27 (type of *P. theicola*) | KC343107 | KC343349 | KC343591 | KC343833 | KC344075 |
| *Diaporthe ganjae* | CBS 180.91 = ILLS 43621 (ex-type) | KC343112 | KC343354 | KC343596 | KC343838 | KC344080 |
| *Diaporthe gardeniae* | CBS 288.56 | KC343113 | KC343355 | KC343597 | KC343839 | KC344081 |
| *Diaporthe helianthi* | CBS 592.81 (paratype) | KC343115 | KC343357 | KC343599 | KC343841 | KC344083 |
|  | CBS 344.94 | KC343114 | KC343356 | KC343598 | KC343840 | KC344082 |
| *Diaporthe hickoriae* | CBS 145.26 (type) | KC343118 | KC343360 | KC343602 | KC343844 | KC344086 |
| *Diaporthe hongkongensis* | CBS 115448 (holotype) = HKUCC 9104 = AT 646 DF 24 (ex-type) | KC343119 | KC343361 | KC343603 | KC343845 | KC344087 |
| *Diaporthe hordei* | CBS 481.92 | KC343120 | KC343362 | KC343604 | KC343846 | KC344088 |
| *Diaporthe impulsa* | CBS 114434 = UPSC 3052 | KC343121 | KC343363 | KC343605 | KC343847 | KC344089 |
|  | CBS 141.27 | KC343122 | KC343364 | KC343606 | KC343848 | KC344090 |
| *Diaporthe inconspicua* | CBS 133813 (type) = LGMF930 = CPC 20306 (ex-type) | KC343123 | KC343365 | KC343607 | KC343849 | KC344091 |
|  | LGMF922 = CPC 20298 | KC343124 | KC343366 | KC343608 | KC343850 | KC344092 |
| *Diaporthe infecunda* | CBS 133812 (type) = LGMF906 = CPC 20282 (ex-type) | KC343126 | KC343368 | KC343610 | KC343852 | KC344094 |
|  | LGMF933 = CPC 20309 | KC343132 | KC343374 | KC343616 | KC343858 | KC344100 |
| *Diaporthe longispora* | CBS 194.36 (type of *D. strumella* var. *longispora* | KC343135 | KC343377 | KC343619 | KC343861 | KC344103 |
| *Diaporthe lusitanicae* | CBS 123212 (holotype) = Di-C001/5 (ex-type) | KC343136 | KC343378 | KC343620 | KC343862 | KC344104 |
|  | CBS 123213 = Di-C001/3 | KC343137 | KC343379 | KC343621 | KC343863 | KC344105 |
| *Diaporthe manihotia* | CBS 505.76 | KC343138 | KC343380 | KC343622 | KC343864 | KC344106 |
| *Diaporthe mayteni* | CBS 133185 = LGMF938 = CPC 20314 (ex-type) | KC343139 | KC343381 | KC343623 | KC343865 | KC344107 |
| *Diaporthe megalospora* | CBS 143.27 | KC343140 | KC343382 | KC343624 | KC343866 | KC344108 |
| *Diaporthe melonis* | CBS 507.78 (isotype) | KC343142 | KC343384 | KC343626 | KC343868 | KC344110 |
|  | CBS 435.87 | KC343141 | KC343383 | KC343625 | KC343867 | KC344109 |
| *Diaporthe musigena* | CBS 129519 (holotype) = CPC 17026 (ex-type) | KC343143 | KC343385 | KC343627 | KC343869 | KC344111 |
| *Diaporthe neilliae* | CBS 144.27 | KC343144 | KC343386 | KC343628 | KC343870 | KC344112 |
| *Diaporthe neoarctii* | CBS 109490 = GB 6421 = AR 3450 (ex-type) | KC343145 | KC343387 | KC343629 | KC343871 | KC344113 |
| *Diaporthe nomurai* | CBS 157.29 | KC343154 | KC343396 | KC343638 | KC343880 | KC344122 |
| *Diaporthe novem* | CBS 127270 (ex-holotype) = 4-27/3-1 (ex-type) | KC343156 | KC343398 | KC343640 | KC343882 | KC344124 |
|  | CBS 354.71 | KC343158 | KC343400 | KC343642 | KC343884 | KC344126 |
| *Diaporthe oncostoma* | CBS 109741 = AR 3445 | KC343161 | KC343403 | KC343645 | KC343887 | KC344129 |
|  | CBS 100454 | KC343160 | KC343402 | KC343644 | KC343886 | KC344128 |
| *Diaporthe oxe* | CBS 133186 ( holotype) = LGMF942 = CPC 20318 (ex-type) | KC343164 | KC343406 | KC343648 | KC343890 | KC344132 |
|  | CBS 133187 = LGMF936 = CPC 20312 | KC343165 | KC343407 | KC343649 | KC343891 | KC344133 |
| *Diaporthe padi* var. *padi* | CBS 114200; UPSC 2569 | KC343169 | KC343411 | KC343653 | KC343895 | KC344137 |
|  | CBS 114649; UPSC 3496 | KC343170 | KC343412 | KC343654 | KC343896 | KC344138 |
| *Diaporthe paranensis* | CBS 133184 (holotype) = LGMF929 = CPC 20305 (ex-type) | KC343171 | KC343413 | KC343655 | KC343897 | KC344139 |
| *Diaporthe perjuncta* | CBS 109745 = ARSEF 3461 = AR 3461 (ex-epitype) | KC343172 | KC343414 | KC343656 | KC343898 | KC344140 |
| *Diaporthe perseae* | CBS 151.73 | KC343173 | KC343415 | KC343657 | KC343899 | KC344141 |
| *Diaporthe phaseolorum* | CBS 116019 = STAM 30 | KC343175 | KC343417 | KC343659 | KC343901 | KC344143 |
|  | CBS 116020; STAM 31 | KC343176 | KC343418 | KC343660 | KC343902 | KC344144 |
| *Diaporthe pseudomangiferae* | CBS 101339 (holotype) | KC343181 | KC343423 | KC343665 | KC343907 | KC344149 |
|  | CBS 388.89 | KC343182 | KC343424 | KC343666 | KC343908 | KC344150 |
| *Diaporthe pseudophoenicicola* | CBS 462.69 (ex-type) | KC343184 | KC343426 | KC343668 | KC343910 | KC344152 |
|  | CBS 176.77 | KC343183 | KC343425 | KC343667 | KC343909 | KC344151 |
| *Diaporthe pustulata* | CBS 109784 = AR 3419 | KC343187 | KC343429 | KC343671 | KC343913 | KC344155 |
|  | CBS 109742 = AR 3430 | KC343185 | KC343427 | KC343669 | KC343911 | KC344153 |
| *Diaporthe raonikayaporum* | CBS 133182 (holotype) = LGMF923 = CPC 20299 (ex-type) | KC343188 | KC343430 | KC343672 | KC343914 | KC344156 |
| *Diaporthe rhoina* | CBS 146.27 | KC343189 | KC343431 | KC343673 | KC343915 | KC344157 |
| *Diaporthe saccarata* | CBS 116311 = STE-U 3743 = CPC 3743 (ex-type) | KC343190 | KC343432 | KC343674 | KC343916 | KC344158 |
| *Diaporthe schini* | CBS 133181 (holotype) = LGMF921 = CPC 20297 (ex-type) | KC343191 | KC343433 | KC343675 | KC343917 | KC344159 |
|  | LGMF910 = CPC 20286 | KC343192 | KC343434 | KC343676 | KC343918 | KC344160 |
| *Diaporthe sclerotioides* | CBS 296.67 (type of *P. sclerotioides*) = ATCC 18585 = IMI 151828 (ex-type) | KC343193 | KC343435 | KC343677 | KC343919 | KC344161 |
|  | CBS 710.76 = PD 76/674 | KC343194 | KC343436 | KC343678 | KC343920 | KC344162 |
| *Diaporthe scobina* | CBS 251.38 | KC343195 | KC343437 | KC343679 | KC343921 | KC344163 |
| *Diaporthe sojae* | CBS 100.87 | KC343196 | KC343438 | KC343680 | KC343922 | KC344164 |
|  | CBS 127267 | HM347700 | KC343441 | KC343683 | HM347685 | KC344167 |
|  | CBS 116017 = DP 0508 = STAM 28 | KC343197 | KC343439 | KC343681 | KC343923 | KC344165 |
|  | CBS 180.55 = ATCC 12050 = CECT 2024 = Alfaro 245 | KC343200 | KC343442 | KC343684 | KC343926 | KC344168 |
| *Diaporthe stictica* | CBS 370.54 | KC343212 | KC343454 | KC343696 | KC343938 | KC344180 |
| *Diaporthe subordinaria* | CBS 101711 | KC343213 | KC343455 | KC343697 | KC343939 | KC344181 |
|  | CBS 464.90 | KC343214 | KC343456 | KC343698 | KC343940 | KC344182 |
| *Diaporthe tecomae* | CBS 100547 | KC343215 | KC343457 | KC343699 | KC343941 | KC344183 |
| *Diaporthe terebinthifolii* | CBS 133180 (holotype) = LGMF914 = CPC 20290 (ex-type) | KC343216 | KC343458 | KC343700 | KC343942 | KC344184 |
|  | LGMF907 = CPC 20283 | KC343217 | KC343459 | KC343701 | KC343943 | KC344185 |
| *Diaporthe toxica* | CBS 534.93 = ATCC 96741 (ex-type) | KC343220 | KC343462 | KC343704 | KC343946 | KC344188 |
|  | CBS 535.93 | KC343221 | KC343463 | KC343705 | KC343947 | KC344189 |
| *Diaporthe vaccinii* | CBS 160.32 (type) = IFO 32646 (ex-type) | KC343228 | KC343470 | KC343712 | KC343954 | KC344196 |
|  | CBS 122112 = FAU 474 | KC343224 | KC343466 | KC343708 | KC343950 | KC344192 |
| *Diaporthe vexans* | CBS 127.14 | KC343229 | KC343471 | KC343713 | KC343955 | KC344197 |
| *Diaporthe viticola* | CBS 113201 (epitype) = STE-U 5683 = CPC 5683 (ex-epitype) | KC343234 | KC343476 | KC343718 | KC343960 | KC344202 |
|  | CBS 109768 = AR 3478 | KC343233 | KC343475 | KC343717 | KC343959 | KC344201 |
| *Diaporthe woodii* | CBS 558.93 | KC343244 | KC343486 | KC343728 | KC343970 | KC344212 |
| *Diaporthe woolworthii* | CBS 148.27 | KC343245 | KC343487 | KC343729 | KC343971 | KC344213 |
| *Diaporthe* cf. *heveae1* | CBS 852.97 | KC343116 | KC343358 | KC343600 | KC343842 | KC344084 |
| *Diaporthe* cf. *heveae2* | CBS 681.84 | KC343117 | KC343359 | KC343601 | KC343843 | KC344085 |
| *Diaporthe sp. 1* | CBS 119639; B 11861 | KC343202 | KC343444 | KC343686 | KC343928 | KC344170 |
|  | LGMF947; CPC 20323 | KC343203 | KC343445 | KC343687 | KC343929 | KC344171 |
| *Diaporthe sp. 2* | LGMF932; CPC 20308 | KC343204 | KC343446 | KC343688 | KC343930 | KC344172 |
| *Diaporthe sp. 3* | CBS 287.29 | KC343205 | KC343447 | KC343689 | KC343931 | KC344173 |
| *Diaporthe sp. 4* | LGMF944; CPC 20320 | KC343206 | KC343448 | KC343690 | KC343932 | KC344174 |
| *Diaporthe sp. 5* | CBS 125575 | KC343207 | KC343449 | KC343691 | KC343933 | KC344175 |
| *Diaporthe sp. 6* | CBS 115584; HKUCC 7784; AT 7 | KC343208 | KC343450 | KC343692 | KC343934 | KC344176 |
|  | CBS 115595; HKUCC 10129 | KC343209 | KC343451 | KC343693 | KC343935 | KC344177 |
| *Diaporthe sp. 7* | CBS 458.78 | KC343210 | KC343452 | KC343694 | KC343936 | KC344178 |
| *Diaporthe sp. 8* | LGMF925; CPC 20301 | KC343211 | KC343453 | KC343695 | KC343937 | KC344179 |
| *Diaporthella corylina* | CBS 121124 = AR 4131 | KC343004 | KC343246 | KC343488 | KC343730 | KC343972 |
