# Supplementary material for: Evaluating multi-locus phylogenies for species boundaries determination in the genus Diaporthe
Source: PeerJ. 2017 Mar 28;5:e3120. doi: 10.7717/peerj.3120 (PMC5372842; doi:10.7717/peerj.3120)
Supplement: Table S2 [file peerj-05-3120-s002.docx]

|  | ITS | TEF1 | TUB | HIS | CAL | ITS TEF1 | ITS TUB | ITS HIS | ITS CAL | TEF1 TUB | TEF1 HIS | TEF1 CAL | TUB HIS | TUB CAL | HIS CAL | ITS TEF1 TUB | ITS TEF1 HIS | ITS TEF1 CAL | ITS TUB HIS | ITS TUB CAL | ITS HIS CAL | TEF1 TUB HIS | TEF1 TUB CAL | TEF1 HIS CAL | TUB HIS CAL | ITS TEF1 TUB HIS | ITS TEF1 TUB CAL | ITS TEF1 HIS CAL | ITS TUB HIS CAL | TEF1 TUB HIS CAL | ITS TEF1 TUB HIS CAL |
| --- | --- | --- | --- | --- | --- | --- | --- | --- | --- | --- | --- | --- | --- | --- | --- | --- | --- | --- | --- | --- | --- | --- | --- | --- | --- | --- | --- | --- | --- | --- | --- |
| ITS | 0 | 51 | 34 | 37 | 47 | 40 | 32 | 28 | 34 | 52 | 52 | 53 | 47 | 56 | 52 | 53 | 46 | 56 | 46 | 48 | 40 | 59 | 62 | 54 | 60 | 58 | 58 | 53 | 56 | 63 | 63 |
| TEF1 | 51 | 0 | 43 | 44 | 46 | 19 | 43 | 45 | 37 | 19 | 15 | 18 | 46 | 41 | 39 | 26 | 25 | 27 | 47 | 35 | 33 | 26 | 31 | 23 | 43 | 27 | 31 | 28 | 41 | 32 | 36 |
| TUB | 34 | 43 | 0 | 37 | 45 | 40 | 22 | 34 | 38 | 32 | 44 | 45 | 21 | 30 | 42 | 35 | 44 | 50 | 28 | 32 | 38 | 33 | 40 | 46 | 34 | 36 | 42 | 49 | 38 | 39 | 43 |
| HIS | 37 | 44 | 37 | 0 | 48 | 43 | 41 | 27 | 41 | 49 | 35 | 52 | 32 | 55 | 41 | 52 | 43 | 57 | 37 | 49 | 39 | 50 | 61 | 47 | 53 | 51 | 57 | 52 | 51 | 58 | 54 |
| CAL | 47 | 46 | 45 | 48 | 0 | 41 | 47 | 49 | 23 | 47 | 47 | 38 | 54 | 39 | 33 | 50 | 49 | 47 | 53 | 37 | 39 | 54 | 45 | 37 | 45 | 53 | 47 | 42 | 43 | 46 | 50 |
| ITS TEF1 | 40 | 19 | 40 | 43 | 41 | 0 | 32 | 30 | 28 | 24 | 28 | 27 | 45 | 42 | 44 | 17 | 14 | 26 | 38 | 32 | 26 | 31 | 34 | 30 | 46 | 28 | 30 | 29 | 40 | 37 | 35 |
| ITS TUB | 32 | 43 | 22 | 41 | 47 | 32 | 0 | 26 | 30 | 34 | 44 | 45 | 33 | 36 | 46 | 29 | 36 | 42 | 22 | 28 | 30 | 39 | 42 | 48 | 42 | 34 | 38 | 43 | 36 | 45 | 39 |
| ITS HIS | 28 | 45 | 34 | 27 | 49 | 30 | 26 | 0 | 30 | 46 | 44 | 45 | 37 | 50 | 44 | 43 | 30 | 46 | 24 | 40 | 28 | 49 | 54 | 48 | 48 | 44 | 50 | 43 | 46 | 51 | 49 |
| ITS CAL | 34 | 37 | 38 | 41 | 23 | 28 | 30 | 30 | 0 | 38 | 36 | 31 | 43 | 34 | 28 | 33 | 30 | 32 | 32 | 24 | 16 | 43 | 38 | 34 | 38 | 36 | 34 | 29 | 32 | 41 | 39 |
| TEF1 TUB | 52 | 19 | 32 | 49 | 47 | 24 | 34 | 46 | 38 | 0 | 28 | 25 | 37 | 30 | 38 | 17 | 28 | 30 | 40 | 28 | 34 | 15 | 18 | 32 | 32 | 20 | 22 | 33 | 34 | 23 | 25 |
| TEF1 HIS | 52 | 15 | 44 | 35 | 47 | 28 | 44 | 44 | 36 | 28 | 0 | 25 | 37 | 38 | 30 | 33 | 20 | 32 | 42 | 32 | 28 | 25 | 36 | 18 | 36 | 30 | 36 | 25 | 34 | 31 | 33 |
| TEF1 CAL | 53 | 18 | 45 | 52 | 38 | 27 | 45 | 45 | 31 | 25 | 25 | 0 | 48 | 29 | 27 | 28 | 29 | 17 | 45 | 23 | 27 | 34 | 17 | 13 | 31 | 29 | 19 | 18 | 29 | 22 | 26 |
| TUB HIS | 47 | 46 | 21 | 32 | 54 | 45 | 33 | 37 | 43 | 37 | 37 | 48 | 0 | 35 | 37 | 40 | 39 | 51 | 21 | 35 | 33 | 30 | 43 | 43 | 27 | 33 | 43 | 44 | 33 | 36 | 38 |
| TUB CAL | 56 | 41 | 30 | 55 | 39 | 42 | 36 | 50 | 34 | 30 | 38 | 29 | 35 | 0 | 28 | 33 | 40 | 32 | 34 | 14 | 30 | 29 | 16 | 28 | 14 | 30 | 24 | 27 | 14 | 19 | 23 |
| HIS CAL | 52 | 39 | 42 | 41 | 33 | 44 | 46 | 44 | 28 | 38 | 30 | 27 | 37 | 28 | 0 | 43 | 34 | 36 | 38 | 28 | 20 | 37 | 30 | 22 | 20 | 38 | 34 | 27 | 24 | 29 | 31 |
| ITS TEF1 TUB | 53 | 26 | 35 | 52 | 50 | 17 | 29 | 43 | 33 | 17 | 33 | 28 | 40 | 33 | 43 | 0 | 23 | 23 | 31 | 23 | 31 | 20 | 23 | 35 | 37 | 13 | 15 | 30 | 31 | 26 | 20 |
| ITS TEF1 HIS | 46 | 25 | 44 | 43 | 49 | 14 | 36 | 30 | 30 | 28 | 20 | 29 | 39 | 40 | 34 | 23 | 0 | 26 | 32 | 30 | 20 | 25 | 34 | 24 | 36 | 24 | 32 | 21 | 32 | 33 | 29 |
| ITS TEF1 CAL | 56 | 27 | 50 | 57 | 47 | 26 | 42 | 46 | 32 | 30 | 32 | 17 | 51 | 32 | 36 | 23 | 26 | 0 | 44 | 20 | 26 | 35 | 22 | 22 | 36 | 26 | 12 | 11 | 28 | 29 | 19 |
| ITS TUB HIS | 46 | 47 | 28 | 37 | 53 | 38 | 22 | 24 | 32 | 40 | 42 | 45 | 21 | 34 | 38 | 31 | 32 | 44 | 0 | 26 | 24 | 31 | 42 | 46 | 30 | 26 | 36 | 41 | 26 | 37 | 33 |
| ITS TUB CAL | 48 | 35 | 32 | 49 | 37 | 32 | 28 | 40 | 24 | 28 | 32 | 23 | 35 | 14 | 28 | 23 | 30 | 20 | 26 | 0 | 22 | 29 | 20 | 24 | 20 | 22 | 14 | 19 | 10 | 21 | 15 |
| ITS HIS CAL | 40 | 33 | 38 | 39 | 39 | 26 | 30 | 28 | 16 | 34 | 28 | 27 | 33 | 30 | 20 | 31 | 20 | 26 | 24 | 22 | 0 | 35 | 34 | 28 | 26 | 30 | 30 | 21 | 22 | 35 | 31 |
| TEF1 TUB HIS | 59 | 26 | 33 | 50 | 54 | 31 | 39 | 49 | 43 | 15 | 25 | 34 | 30 | 29 | 37 | 20 | 25 | 35 | 31 | 29 | 35 | 0 | 25 | 33 | 25 | 9 | 25 | 34 | 27 | 18 | 20 |
| TEF1 TUB CAL | 62 | 31 | 40 | 61 | 45 | 34 | 42 | 54 | 38 | 18 | 36 | 17 | 43 | 16 | 30 | 23 | 34 | 22 | 42 | 20 | 34 | 25 | 0 | 24 | 22 | 22 | 12 | 23 | 22 | 11 | 15 |
| TEF1 HIS CAL | 54 | 23 | 46 | 47 | 37 | 30 | 48 | 48 | 34 | 32 | 18 | 13 | 43 | 28 | 22 | 35 | 24 | 22 | 46 | 24 | 28 | 33 | 24 | 0 | 26 | 34 | 28 | 13 | 26 | 23 | 27 |
| TUB HIS CAL | 60 | 43 | 34 | 53 | 45 | 46 | 42 | 48 | 38 | 32 | 36 | 31 | 27 | 14 | 20 | 37 | 36 | 36 | 30 | 20 | 26 | 25 | 22 | 26 | 0 | 28 | 28 | 29 | 10 | 17 | 23 |
| ITS TEF1 TUB HIS | 58 | 27 | 36 | 51 | 53 | 28 | 34 | 44 | 36 | 20 | 30 | 29 | 33 | 30 | 38 | 13 | 24 | 26 | 26 | 22 | 30 | 9 | 22 | 34 | 28 | 0 | 16 | 29 | 22 | 19 | 15 |
| ITS TEF1 TUB CAL | 58 | 31 | 42 | 57 | 47 | 30 | 38 | 50 | 34 | 22 | 36 | 19 | 43 | 24 | 34 | 15 | 32 | 12 | 36 | 14 | 30 | 25 | 12 | 28 | 28 | 16 | 0 | 21 | 20 | 17 | 7 |
| ITS TEF1 HIS CAL | 53 | 28 | 49 | 52 | 42 | 29 | 43 | 43 | 29 | 33 | 25 | 18 | 44 | 27 | 27 | 30 | 21 | 11 | 41 | 19 | 21 | 34 | 23 | 13 | 29 | 29 | 21 | 0 | 21 | 26 | 20 |
| ITS TUB HIS CAL | 56 | 41 | 38 | 51 | 43 | 40 | 36 | 46 | 32 | 34 | 34 | 29 | 33 | 14 | 24 | 31 | 32 | 28 | 26 | 10 | 22 | 27 | 22 | 26 | 10 | 22 | 20 | 21 | 0 | 19 | 15 |
| TEF1 TUB HIS CAL | 63 | 32 | 39 | 58 | 46 | 37 | 45 | 51 | 41 | 23 | 31 | 22 | 36 | 19 | 29 | 26 | 33 | 29 | 37 | 21 | 35 | 18 | 11 | 23 | 17 | 19 | 17 | 26 | 19 | 0 | 14 |
| ITS TEF1 TUB HIS CAL | 63 | 36 | 43 | 54 | 50 | 35 | 39 | 49 | 39 | 25 | 33 | 26 | 38 | 23 | 31 | 20 | 29 | 19 | 33 | 15 | 31 | 20 | 15 | 27 | 23 | 15 | 7 | 20 | 15 | 14 | 0 |
