# Supplementary material for: Evaluating multi-locus phylogenies for species boundaries determination in the genus Diaporthe
Source: PeerJ. 2017 Mar 28;5:e3120. doi: 10.7717/peerj.3120 (PMC5372842; doi:10.7717/peerj.3120)
Supplement: Table S3 [file peerj-05-3120-s003.docx]

|  | ITS | TEF1 | TUB | HIS | CAL | ITS TEF1 | ITS TUB | ITS HIS | ITS CAL | TEF1 TUB | TEF1 HIS | TEF1 CAL | TUB HIS | TUB CAL | HIS CAL | ITS TEF1 TUB | ITS TEF1 HIS | ITS TEF1 CAL | ITS TUB HIS | ITS TUB CAL | ITS HIS CAL | TEF1 TUB HIS | TEF1 TUB CAL | TEF1 HIS CAL | TUB HIS CAL | ITS TEF1 TUB HIS | ITS TEF1 TUB CAL | ITS TEF1 HIS CAL | ITS TUB HIS CAL | TEF1 TUB HIS CAL | ITS TEF1 TUB HIS CAL |
| --- | --- | --- | --- | --- | --- | --- | --- | --- | --- | --- | --- | --- | --- | --- | --- | --- | --- | --- | --- | --- | --- | --- | --- | --- | --- | --- | --- | --- | --- | --- | --- |
| ITS | 0 | 45 | 46 | 32 | 46 | 50 | 43 | 37 | 46 | 65 | 52 | 54 | 55 | 61 | 53 | 56 | 53 | 56 | 47 | 52 | 48 | 69 | 66 | 58 | 66 | 64 | 70 | 58 | 58 | 68 | 66 |
| TEF1 | 45 | 0 | 37 | 35 | 33 | 19 | 28 | 38 | 25 | 26 | 13 | 15 | 34 | 34 | 28 | 21 | 20 | 19 | 28 | 31 | 29 | 32 | 27 | 21 | 35 | 27 | 31 | 23 | 33 | 31 | 31 |
| TUB | 46 | 37 | 0 | 30 | 38 | 38 | 19 | 35 | 36 | 29 | 38 | 38 | 19 | 25 | 47 | 28 | 41 | 44 | 21 | 24 | 38 | 31 | 38 | 42 | 32 | 34 | 40 | 44 | 30 | 34 | 38 |
| HIS | 32 | 35 | 30 | 0 | 34 | 42 | 31 | 17 | 38 | 49 | 38 | 42 | 37 | 47 | 37 | 42 | 39 | 48 | 29 | 40 | 36 | 51 | 54 | 46 | 50 | 46 | 56 | 48 | 44 | 54 | 52 |
| CAL | 46 | 33 | 38 | 34 | 0 | 40 | 35 | 41 | 26 | 41 | 34 | 26 | 45 | 31 | 23 | 38 | 39 | 34 | 39 | 28 | 24 | 43 | 36 | 30 | 34 | 40 | 40 | 36 | 36 | 40 | 40 |
| ITS TEF1 | 50 | 19 | 38 | 42 | 40 | 0 | 25 | 31 | 26 | 25 | 16 | 18 | 31 | 37 | 33 | 18 | 13 | 16 | 25 | 32 | 28 | 29 | 26 | 24 | 36 | 26 | 30 | 22 | 32 | 32 | 30 |
| ITS TUB | 43 | 28 | 19 | 31 | 35 | 25 | 0 | 26 | 23 | 28 | 27 | 27 | 18 | 24 | 36 | 17 | 30 | 29 | 12 | 17 | 23 | 32 | 31 | 33 | 27 | 27 | 31 | 31 | 19 | 31 | 29 |
| ITS HIS | 37 | 38 | 35 | 17 | 41 | 31 | 26 | 0 | 33 | 46 | 37 | 39 | 32 | 46 | 38 | 35 | 32 | 41 | 24 | 37 | 33 | 48 | 49 | 45 | 47 | 41 | 51 | 45 | 39 | 49 | 47 |
| ITS CAL | 46 | 25 | 36 | 38 | 26 | 26 | 23 | 33 | 0 | 33 | 24 | 18 | 33 | 25 | 21 | 24 | 29 | 18 | 25 | 20 | 10 | 35 | 26 | 24 | 28 | 28 | 30 | 22 | 22 | 30 | 30 |
| TEF1 TUB | 65 | 26 | 29 | 49 | 41 | 25 | 28 | 46 | 33 | 0 | 23 | 21 | 26 | 24 | 38 | 11 | 28 | 25 | 26 | 27 | 35 | 10 | 11 | 25 | 25 | 13 | 17 | 27 | 27 | 21 | 17 |
| TEF1 HIS | 52 | 13 | 38 | 38 | 34 | 16 | 27 | 37 | 24 | 23 | 0 | 12 | 29 | 33 | 25 | 20 | 11 | 16 | 25 | 30 | 24 | 21 | 24 | 12 | 30 | 18 | 28 | 14 | 28 | 22 | 22 |
| TEF1 CAL | 54 | 15 | 38 | 42 | 26 | 18 | 27 | 39 | 18 | 21 | 12 | 0 | 33 | 23 | 19 | 18 | 21 | 8 | 29 | 22 | 18 | 25 | 14 | 8 | 22 | 22 | 18 | 12 | 22 | 20 | 22 |
| TUB HIS | 55 | 34 | 19 | 37 | 45 | 31 | 18 | 32 | 33 | 26 | 29 | 33 | 0 | 28 | 40 | 23 | 30 | 37 | 8 | 27 | 33 | 24 | 33 | 35 | 25 | 25 | 35 | 35 | 23 | 29 | 31 |
| TUB CAL | 61 | 34 | 25 | 47 | 31 | 37 | 24 | 46 | 25 | 24 | 33 | 23 | 28 | 0 | 30 | 23 | 40 | 27 | 28 | 9 | 27 | 26 | 19 | 27 | 13 | 27 | 23 | 29 | 21 | 19 | 27 |
| HIS CAL | 53 | 28 | 47 | 37 | 23 | 33 | 36 | 38 | 21 | 38 | 25 | 19 | 40 | 30 | 0 | 33 | 32 | 25 | 36 | 29 | 17 | 40 | 29 | 21 | 21 | 35 | 33 | 25 | 29 | 31 | 33 |
| ITS TEF1 TUB | 56 | 21 | 28 | 42 | 38 | 18 | 17 | 35 | 24 | 11 | 20 | 18 | 23 | 23 | 33 | 0 | 27 | 18 | 17 | 16 | 26 | 17 | 16 | 24 | 24 | 14 | 16 | 22 | 18 | 20 | 18 |
| ITS TEF1 HIS | 53 | 20 | 41 | 39 | 39 | 13 | 30 | 32 | 29 | 28 | 11 | 21 | 30 | 40 | 32 | 27 | 0 | 21 | 26 | 35 | 29 | 26 | 33 | 21 | 37 | 19 | 35 | 19 | 33 | 31 | 27 |
| ITS TEF1 CAL | 56 | 19 | 44 | 48 | 34 | 16 | 29 | 41 | 18 | 25 | 16 | 8 | 37 | 27 | 25 | 18 | 21 | 0 | 31 | 24 | 20 | 29 | 16 | 12 | 26 | 22 | 18 | 8 | 24 | 22 | 22 |
| ITS TUB HIS | 47 | 28 | 21 | 29 | 39 | 25 | 12 | 24 | 25 | 26 | 25 | 29 | 8 | 28 | 36 | 17 | 26 | 31 | 0 | 19 | 25 | 26 | 31 | 31 | 27 | 21 | 31 | 29 | 17 | 29 | 27 |
| ITS TUB CAL | 52 | 31 | 24 | 40 | 28 | 32 | 17 | 37 | 20 | 27 | 30 | 22 | 27 | 9 | 29 | 16 | 35 | 24 | 19 | 0 | 20 | 27 | 22 | 26 | 18 | 24 | 22 | 26 | 14 | 22 | 26 |
| ITS HIS CAL | 48 | 29 | 38 | 36 | 24 | 28 | 23 | 33 | 10 | 35 | 24 | 18 | 33 | 27 | 17 | 26 | 29 | 20 | 25 | 20 | 0 | 37 | 28 | 24 | 26 | 30 | 32 | 22 | 16 | 32 | 32 |
| TEF1 TUB HIS | 69 | 32 | 31 | 51 | 43 | 29 | 32 | 48 | 35 | 10 | 21 | 25 | 24 | 26 | 40 | 17 | 26 | 29 | 26 | 27 | 37 | 0 | 19 | 25 | 25 | 9 | 21 | 27 | 27 | 17 | 15 |
| TEF1 TUB CAL | 66 | 27 | 38 | 54 | 36 | 26 | 31 | 49 | 26 | 11 | 24 | 14 | 33 | 19 | 29 | 16 | 33 | 16 | 31 | 22 | 28 | 19 | 0 | 16 | 18 | 20 | 10 | 18 | 24 | 14 | 20 |
| TEF1 HIS CAL | 58 | 21 | 42 | 46 | 30 | 24 | 33 | 45 | 24 | 25 | 12 | 8 | 35 | 27 | 21 | 24 | 21 | 12 | 31 | 26 | 24 | 25 | 16 | 0 | 22 | 22 | 16 | 6 | 22 | 16 | 18 |
| TUB HIS CAL | 66 | 35 | 32 | 50 | 34 | 36 | 27 | 47 | 28 | 25 | 30 | 22 | 25 | 13 | 21 | 24 | 37 | 26 | 27 | 18 | 26 | 25 | 18 | 22 | 0 | 24 | 22 | 24 | 18 | 14 | 22 |
| ITS TEF1 TUB HIS | 64 | 27 | 34 | 46 | 40 | 26 | 27 | 41 | 28 | 13 | 18 | 22 | 25 | 27 | 35 | 14 | 19 | 22 | 21 | 24 | 30 | 9 | 20 | 22 | 24 | 0 | 18 | 18 | 22 | 18 | 10 |
| ITS TEF1 TUB CAL | 70 | 31 | 40 | 56 | 40 | 30 | 31 | 51 | 30 | 17 | 28 | 18 | 35 | 23 | 33 | 16 | 35 | 18 | 31 | 22 | 32 | 21 | 10 | 16 | 22 | 18 | 0 | 16 | 22 | 14 | 12 |
| ITS TEF1 HIS CAL | 58 | 23 | 44 | 48 | 36 | 22 | 31 | 45 | 22 | 27 | 14 | 12 | 35 | 29 | 25 | 22 | 19 | 8 | 29 | 26 | 22 | 27 | 18 | 6 | 24 | 18 | 16 | 0 | 20 | 16 | 16 |
| ITS TUB HIS CAL | 58 | 33 | 30 | 44 | 36 | 32 | 19 | 39 | 22 | 27 | 28 | 22 | 23 | 21 | 29 | 18 | 33 | 24 | 17 | 14 | 16 | 27 | 24 | 22 | 18 | 22 | 22 | 20 | 0 | 22 | 22 |
| TEF1 TUB HIS CAL | 68 | 31 | 34 | 54 | 40 | 32 | 31 | 49 | 30 | 21 | 22 | 20 | 29 | 19 | 31 | 20 | 31 | 22 | 29 | 22 | 32 | 17 | 14 | 16 | 14 | 18 | 14 | 16 | 22 | 0 | 12 |
| ITS TEF1 TUB HIS CAL | 66 | 31 | 38 | 52 | 40 | 30 | 29 | 47 | 30 | 17 | 22 | 22 | 31 | 27 | 33 | 18 | 27 | 22 | 27 | 26 | 32 | 15 | 20 | 18 | 22 | 10 | 12 | 16 | 22 | 12 | 0 |
