# Supplementary figures and images for: Evaluating multi-locus phylogenies for species boundaries determination in the genus Diaporthe
Source: PeerJ. 2017 Mar 28;5:e3120. doi: 10.7717/peerj.3120 (PMC5372842; doi:10.7717/peerj.3120)

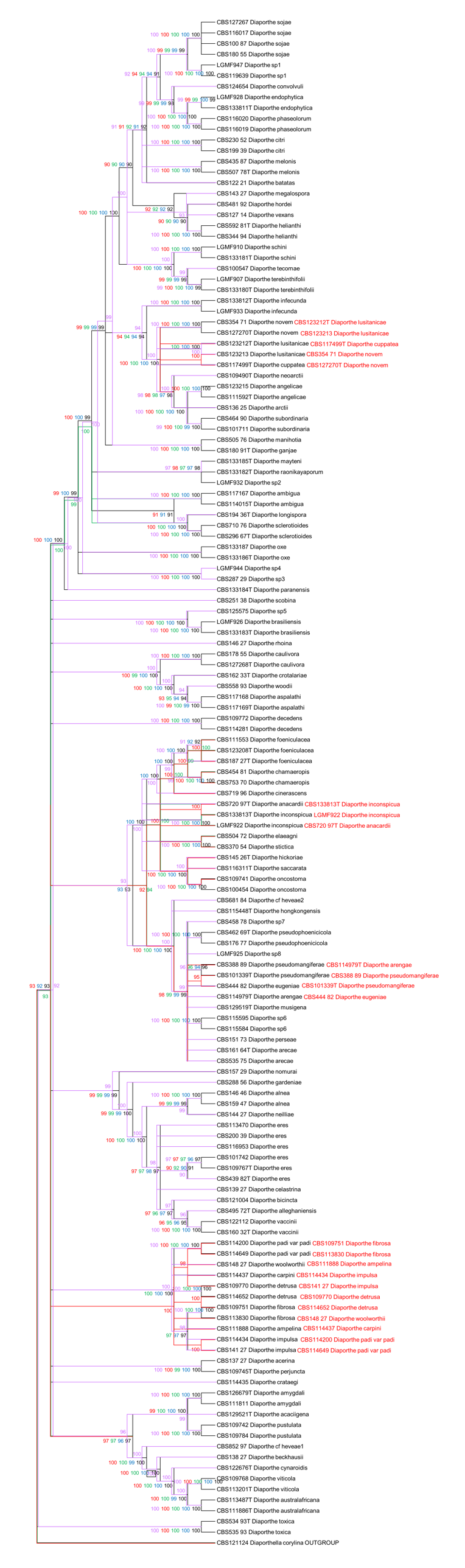

Supplement: Figure S5 — The numbers represent the bootstrap value for the branching in each tree. Red –MP tree with TEF in the first position of the alignment. Mauve –MP tree with TEF in the second position of the alignment. Green –MP tree with TEF in the third position of the alignment. Blue –MP tree with TEF in the fourth position of the alignment. Black –MP tree with TEF in the fifth position of the alignment. The only small effect of shifting the position of TEF is observed in the TEF-ITS-TUB-HIS-CAL (red) and ITS-TEF-TUB-HIS-CAL (mauve) trees. In TEF-ITS-TUB-HIS-CAL case, flipping is observed in a small number of terminal branches. That flipping does not affect the overall topology of the tree, which is identical to that of the remaining trees. These results suggest that changing the order of the genes in the alignment will have a negligible effect on the topology of the phylogenetic trees. [file peerj-05-3120-s008.png]
